# Implementation strategies to address the determinants of adoption, implementation, and maintenance of a clinical decision support tool for emergency department buprenorphine initiation: a qualitative study

**DOI:** 10.1186/s43058-023-00421-7

**Published:** 2023-04-20

**Authors:** Matthew J. Simpson, Carly Ritger, Jason A. Hoppe, Wesley C. Holland, Megan A. Morris, Bidisha Nath, Edward R. Melnick, Caroline Tietbohl

**Affiliations:** 1 Department of Family Medicine, University of Colorado Anschutz Medical Campus, 12631 E. 17th Avenue, Box F496, Aurora, CO 80045 USA; 2 Adult and Child Center for Health Outcomes Research & Delivery Science, University of Colorado Anschutz Medical Campus, 13199 E. Montview Boulevard, Suite 300, Aurora, CO 80045 USA; 3 Department of Emergency Medicine, University of Colorado Anschutz Medical Campus, 12631 E. 17th Avenue, Box B215, Aurora, CO 80045 USA; 4 Yale University School of Medicine, 333 Cedar St., New Haven, CT 06510 USA; 5 Department of Emergency Medicine, Yale School of Medicine, 464 Congress Ave., Ste 260, New Haven, CT 06519 USA

**Keywords:** Buprenorphine, Opioid use disorder, Implementation science, Emergency medicine

## Abstract

**Background:**

Untreated opioid use disorder (OUD) is a significant public health problem. Buprenorphine is an evidence-based treatment for OUD that can be initiated in and prescribed from emergency departments (EDs) and office settings. Adoption of buprenorphine initiation among ED clinicians is low. The EMBED pragmatic clinical trial investigated the effectiveness of a clinical decision support (CDS) tool to promote ED clinicians’ behavior related to buprenorphine initiation in the ED. While the CDS intervention was not associated with increased rates of buprenorphine treatment for patients with OUD at intervention ED sites, attending physicians at intervention EDs were more likely to initiate buprenorphine at least once over the duration of the study compared to those in the usual care arms (44.4% vs 34.0%, *P* = 0.01). This suggests the CDS intervention may be associated with increased adoption of buprenorphine initiation. As a secondary aim, we sought to identify the determinants of CDS adoption, implementation, and maintenance in a variety of ED settings and geographic locations.

**Methods:**

We purposively sampled and conducted semi-structured, in-depth interviews with clinicians across EMBED trial sites randomized to the intervention arm from five healthcare systems. Interviews elicited clinician experiences regarding buprenorphine initiation and CDS use. Interviews were analyzed using directed content analysis informed by the Practical, Robust Implementation and Sustainability Model (PRISM). We used a hybrid approach (a priori codes informed by PRISM and emergent codes) for codebook development. ATLAS.ti (version 9.0) was used for data management. Coded data were analyzed within individual interview transcripts and across all interviews to identify major themes. This process involved (1) combining, comparing, and making connections between codes; (2) writing analytic memos about observed patterns; and (3) frequent team meetings to discuss emerging patterns.

**Results:**

Twenty-eight interviews were conducted. Major themes that influenced the successful adoption, implementation, and maintenance of the EMBED intervention and ED-initiated BUP were organizational culture and commitment, clinician training and support, the ability to connect patients to ongoing treatment, and the ability to tailor implementation to each ED. These findings informed the identification of implementation strategies (framed using PRISM domains) to enhance the ED initiation of buprenorphine.

**Conclusion:**

The findings from this qualitative analysis can provide guidance to build better systems to promote the adoption of ED-initiated buprenorphine.

**Supplementary Information:**

The online version contains supplementary material available at 10.1186/s43058-023-00421-7.

Contributions to the literature

- Initiation of buprenorphine for opioid use disorder in the emergency department is an evidence-based practice with low adoption among emergency medicine clinicians.
- Clinical decision support embedded in the electronic health record showed a modest increase in clinician adoption of buprenorphine initiation in the emergency department with little impact on patient-level outcomes.
- These findings support the development of comprehensive, multilevel implementation strategies to accompany clinical decision support interventions to achieve a bigger impact.
- Leveraging the PRISM framework, we used qualitative methods to identify key determinants of the adoption, implementation, and maintenance of a clinical decision support intervention for emergency department buprenorphine initiation.

## Introduction

An estimated 2.7 million people in the USA had opioid use disorder (OUD) in 2020, and this number has continued to grow due to the COVID-19 pandemic’s effect and increasing prevalence of highly potent synthetic opioids like fentanyl [1–5]. The emergency department (ED) represents a key opportunity to engage patients in evidence-based treatment for OUD, including buprenorphine (BUP). BUP is a partial opioid agonist medication that is effective at decreasing opioid withdrawal, craving, overdose, and mortality [6–9]. BUP can be safely initiated in the ED, is cost-effective, and is associated with a nearly twofold increase in the probability of remaining engaged in formal addiction treatment following discharge [10, 11]. However, ED-initiated BUP remains underutilized [12]. Previously identified barriers include the complexity involved with initiating BUP (intervention—organizational perspective); stigma; competing priorities in ED settings (recipients—organizational perspective); lack of physician knowledge, training, and preparedness to determine OUD care needs; availability of dedicated team members to support patients with OUD (implementation and sustainability infrastructure); and challenges connecting patients with outpatient treatment (external environment) [13–16].

As electronic health records (EHRs) become ubiquitous, clinical decision support (CDS) can facilitate the adoption of ED-initiated BUP by addressing some known barriers. The EMergency department-initiated BuprenorphinE for opioid use Disorder (EMBED) CDS is a clinician-facing application integrated within the EHR developed through a user-centered design process [17]. The EMBED CDS offers optional evidence-based decision support to assist clinicians with the diagnosis of OUD, assessment of withdrawal severity, and assessment of patient readiness to begin treatment, all of which inform the selection of care options within the CDS. Integrated CDS automates several EHR activities related to initiating BUP, including orders, prescriptions, documentation, discharge instructions, and referral for ongoing treatment [17, 18].

To evaluate CDS effectiveness at increasing rates of ED-initiated BUP for OUD, EMBED was evaluated in a pragmatic, group randomized trial across 21 EDs in five healthcare systems in five states [19, 20]. The parent EMBED study found that the CDS was not associated with increased rates of BUP treatment for patients with OUD at intervention ED sites compared to sites randomized to usual care. However, attending physicians at intervention EDs were more likely to initiate BUP at least once over the duration of the study compared to those in the usual care arms (44.4% vs 34.0%, *P* = 0.01), suggesting the intervention may be associated with increased adoption of this practice. This is consistent with other studies that show that CDS typically has a small effect on patient outcomes when used as a standalone intervention [21]—suggesting that more comprehensive, multilevel strategies are needed for reliable practice improvement and subsequent impact at the patient level. Our overall goal with this study was to identify the determinants and corresponding implementation strategies that influenced the adoption, implementation, and maintenance of a CDS intervention for ED-initiated BUP. Therefore, the objectives of this study were to (1) leverage the Practical, Robust Implementation and Sustainability Model (PRISM) to understand the determinants of adoption, implementation, and maintenance of CDS to support BUP initiation in the ED and (2) identify other key contextual factors in addition to CDS that support increased adoption of ED-initiated BUP.

## Methods

### The EMBED clinical trial

The parent clinical trial was a pragmatic, cluster randomized controlled trial (ClinicalTrials.gov: NCT03658642) that included 18 ED clusters across five healthcare systems in the USA. Sites were randomly assigned to receive the EMBED CDS tool described above [19, 20]. All study sites received education on OUD and BUP initiation. Intervention sites also received training on how to use the EMBED CDS [20]. While a Drug Addiction Treatment Act (DATA) of 2000 waiver was necessary to prescribe BUP to patients upon discharge from the ED during the trial period, [22] clinicians were not required to obtain this waiver as part of the study but could still administer buprenorphine in the ED under the 72-h rule [23].

### Study design and implementation science framework

**Fig. 1:**
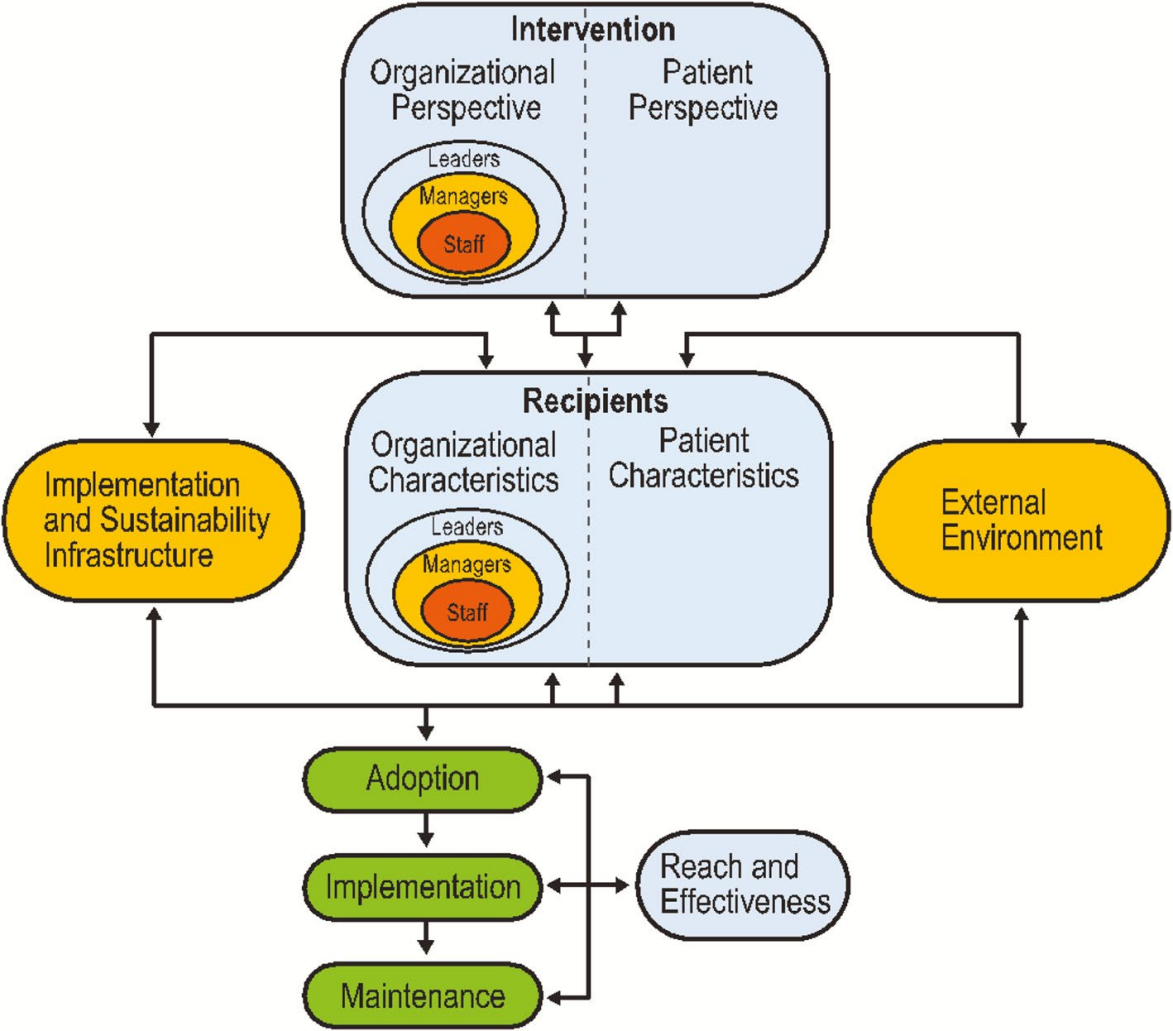
The Practical Robust Implementation Sustainability Model (PRISM)

Guided by the qualitative content analysis methodology, the present study aimed to identify the multilevel determinants that influenced the adoption, implementation, and maintenance of ED-initiated BUP and the use of the EMBED CDS. For this study, determinants were defined as any factor that influenced the adoption, implementation, and maintenance of ED-initiated BUP and use of the EMBED CDS and encompassed barriers and impediments as well as facilitators and enablers [24]. Consistent with established definitions, [25] adoption refers to the willingness to utilize the EMBED intervention, implementation refers to the process of integrating the EMBED intervention into clinical care at each ED, and maintenance refers to the continued use of the EMBED intervention. PRISM (Fig. 1) was selected to guide the research as it was developed to systematically incorporate multilevel factors to plan, evaluate, and disseminate evidence-based interventions [26]. PRISM includes the key domains of Intervention, Recipients, Implementation and Sustainability Infrastructure, and External Environment, which are either known or hypothesized to influence the adoption, implementation, and maintenance of ED-initiated BUP [27]. This manuscript was written in accordance with the Consolidated Criteria for Reporting Qualitative Studies (COREQ) standards [28].

### Settings and participants

Purposive sampling was used to recruit clinicians (1) across all healthcare systems randomized to the intervention arm and (2) with a range of experience with using the EMBED CDS tool. Potential participants were eligible if they were practicing clinicians at an intervention site during the study period and had been willing to use (i.e., adopted) the EMBED CDS tool. Email invites were sent from both the study team and local project leaders. Respondents were screened for inclusion by a research analyst (CR) prior to confirming study eligibility and scheduling an interview.

### Data collection

Our interdisciplinary team developed, pilot tested, and refined a semi-structured interview guide consisting of questions regarding the ED role, practice patterns regarding ED BUP initiation, and utilization of the CDS tool. Probes based on the PRISM framework identified factors that influenced decisions to prescribe BUP or utilize the CDS, including characteristics of the CDS tool, training, organizational characteristics, and external factors. A brief demographic survey was administered after each interview.

Individual interviews took place over Zoom (Zoom Video Communications, San Jose, CA) video conference between November 2021 and March 2022, lasting 45–60 min each. Interviews were audio-recorded with participant permission and professionally transcribed. Interview recruitment continued until no additional, novel information was discussed in interviews; that is, saturation was reached on the topics of factors that led participants to prescribe buprenorphine. All study participants provided informed consent for their participation. The study protocol was approved as exempt from human subjects review by the Colorado Multiple Institutional Review Board.

### Qualitative analysis

The qualitative team included one female master’s degree-prepared qualitative research analyst (CR), one female PhD sociologist (CT), one female PhD qualitative researcher (MM), and one male physician (MS). All members had qualitative methods training and prior experience conducting qualitative research. At least one team member was present at all interviews. The team members had no prior relationship with the participants and explained that their sole purpose was to conduct the interview and glean perspectives on ED-initiated BUP and CDS use.

The analysis team used a directed content analysis [29] approach including both a priori and emergent codes to categorize interview segments. The a priori codes were selected and defined based on the domains and constructs of the PRISM framework [27]. The study team members identified emergent codes by labeling segments of interview text with descriptors that captured any concepts or ideas discussed by the participants that were not already reflected in the PRISM domain codes (e.g., BUP initiation process, stigma around BUP initiation, motivations to use EMBED). The codebook was iteratively updated as new emergent codes were identified, until code saturation was reached [30]. The larger research team met regularly throughout the initial coding process to refine the coding structure and compare the application of codes, thus establishing the trustworthiness of the data. A stable coding structure and application of codes were reached after three interviews, after which the qualitative analyst independently coded the remaining 25 interviews. To ensure ongoing coding consistency, double coding continued intermittently throughout the coding process so that ~ 20% (*N* = 5) of the transcripts were double-coded. Three team members (CR, CT, MS) coded the data. ATLAS.ti version 9 (ATLAS.ti GmbH, Berlin, Germany) was used to facilitate data management. Coded data were analyzed within individual interview transcripts and across all interviews in the dataset to identify major themes. This process involved (1) combining, comparing, and making connections between codes based on a review of coded data reports; (2) writing analytic memos to summarize coded data reports, record salient themes and quotes (including both PRISM-focused reflections and observations that were identified inductively), and describe impressions; and (3) frequent team meetings to discuss reflexivity and emerging patterns.

## Results

Thirty-three clinicians responded to the interview invitations, 31 completed screening for interview eligibility, and 28 interviews were conducted (Table 1). All five participating healthcare systems (1–3 EDs per system) were represented in the final sample. Reasons for not participating included the clinician not working at an intervention site, unfamiliarity with the CDS tool, and lack of response. A majority of those interviewed (*n* = 23) were attending physicians; 3 were physician assistants, and 2 were resident physicians. Years in practice ranged from 3 to 23 years (mean 11.5 years). Of those interviewed, 12 were women and 16 were men. Most interviewees identified as white (*n* = 24).

**Table 1:** Participant characteristics

	Number	Percent
**Role in ED**		
**Physician assistant**	3	10.7
**Resident physician**	2	7.1
**Attending physician**	23	82.1
**Gender**		
**Female**	12	42.9
**Male**	16	57.1
**Race/ethnicity**		
**Non-White**	4	14.3
**White**	24	85.7
	**Mean (years)**	**Range (years)**
**Time in practice**	11.5	3–23
**Time at current ED**	6.75	1–17

The analysis yielded the following four broad categories of factors that influenced the adoption, implementation, and maintenance of the EMBED CDS tool: organizational culture and commitment, clinician training and support, ability to tailor implementation of the CDS tool to the local setting, and the ability to connect patients for ongoing treatment. The corresponding PRISM domains are included within each theme and summarized in Table 2.

**Table 2:** Summary of the multilevel determinants of ED initiation of buprenorphine through integration of the EMBED clinical decision support tool, guided by PRISM

PRISM domain	Determinants
**Intervention (organizational perspective)**	• Clinician level: evidence to support buprenorphine initiation and the EMBED CDS • Clinician level: ability to observe positive results from using the intervention, such finding out when a patient establishes care for OUD treatment after discharge
**Recipients (organizational characteristics)**	• Organizational level: perspective on buprenorphine initiation, including establishing buprenorphine initiation as a cultural norm within the department and communicating that clearly to all frontline clinicians and staff
**Implementation and sustainability infrastructure**	• Organizational level: ability to tailor the intervention to the local environment, including identifying existing interdisciplinary team members in the ED and the organization to implement the intervention, and the ability to integrate the CDS tool within the EHR • Organizational level: commitment to implement and sustain intervention through activities such as designating local champions/experts • Clinician level: sufficient training and support for prescribing clinicians
**External environment**	• Ability to link patients for ongoing treatment

### Organizational culture and commitment

#### Establishing BUP initiation as a cultural norm (recipients—organizational perspective)

Many participants referenced elements of organizational culture and commitment as factors that facilitated their ability to utilize CDS and initiate BUP from the ED. Organizational culture refers to the shared values and norms among clinicians and staff members in the ED, [31] including the belief that caring for patients with OUD is a shared responsibility among the clinical team and initiating BUP for OUD is a supported practice in their ED. Participants often discussed a local culture in their ED in which initiating BUP for patients with OUD is the norm.

Some participants stated that they successfully incorporated BUP initiation into their clinical practice because of a “culture of prescribing” at their institution. Participants who referenced a “culture of prescribing” felt their EDs were open and encouraging of implementing ED-initiated BUP, so it seemed within their scope of practice:

It’s a culture of ‘this is a thing we do.’ You’re not out on a limb. You’re not doing something crazy. Our culture is we can prescribe this. This is one of our tools at our disposal. (Interviewee 8, Site 2)

For some participants, the “culture of prescribing” was reinforced by interactions with colleagues. As one interviewee described, both senior colleagues and residents modeled the adoption of the practice of BUP initiation. Additionally, this participant felt that there was such strong communication around ED-initiated BUP at their ED that it was viewed as a standard practice:

The fact that other attendings who are more senior than me were earlier adopters, more experienced with it [led to] residents…asking to prescribe [BUP]. (Interviewee 20, Site 3)

In contrast, participants at one site described how a less supportive culture for BUP initiation impeded adoption. One participant noted that they “don’t think it [BUP initiation and EMBED] really got a lot of traction” at their hospital (Interviewee 22, Site 4); while the participant reported that they personally perceived prescribing BUP as part of their job, only one other clinician at their site routinely used EMBED. Another clinician at the same site discussed a situation where a patient asked for BUP, but ED staff questioned whether their institution endorsed prescribing it. The interviewee attributed this behavior to OUD stigma and prescribing infrequency for BUP at their site:

I had a guy who came in very agitated, very upset, demanding opioids for treatment because of withdrawal … The patient was asking for buprenorphine, and I think the staff were like, ‘Well, we don’t do that.’ I was like, ‘No, we do. We just don’t often do that, but we can’…I think it’s probably two things: I think it’s either not very common, which is probably true, and I think that there’s some judgement. You are the one that got addicted to X and this is your ‘punishment’ for that. (Interviewee 21, Site 4)

Stigma also seemed to influence adoption at other sites where communication about and prioritization of caring for patients with OUD were not as strong. Members of these clinical teams were unaware of the priority to treat OUD and thus believed it was outside their responsibilities.

#### Organizational commitment to implement and sustain intervention (implementation and sustainability infrastructure)

Establishing BUP initiation as a cultural norm within the ED was often coupled with an organizational commitment to implement and sustain the intervention. Organizational commitment refers to dedicating resources to support caring for patients with OUD, including allocating personnel to manage the implementation and sustainability of EMBED. As one participant explained, their site clinicians could reliably contact someone to help them prescribe:

Having experts in our department who are willing to answer questions, who are accessible and willing to answer questions about it [use of EMBED or BUP initiation] is really helpful. (Interviewee 8, Site 2)

Another example of organizational commitment was the designation of at least one local champion that other clinicians could contact for assistance with BUP initiation and/or the EMBED intervention. Participants at EDs that had a local champion indicated more willingness to initiate BUP. Additionally, participants described how selecting the right person to serve as a local champion could contribute to implementation success:

We do have a very specific colleague who’s a champion, who’s really doing groundbreaking work in this area and makes us…all feel really proud to be on faculty with them… I think also he’s a champion who’s been here for a long time, so he’s respected and people know him. It felt like he was an insider doing this as opposed to, let’s say, somebody who’d just joined the department, was brand new, and then was like, ‘Hey, we’re gonna do this whole thing.’ I think, then, some people might’ve had a response of like, ‘But you don’t even know what it’s like here.’ (Interviewee 4, Site 1)

In contrast, one participant attributed the lack of success of EMBED and BUP initiation at their institution to the absence of a local champion:

If you’re trying to initiate something new, you’d need whatever that person who’s the go-to person, who knows the ins and outs. I don’t know what you want to call it, but the leader or the champion of whatever new process you’re trying to put in place. That’s what would have helped—‘cause I wasn’t that person, and my buddy wasn’t that person either, so we were just out there trying to figure it out. (Interviewee 22, Site 4)

Although this clinician and their colleague were willing to use EMBED and initiate BUP, they were not designated champions for the intervention and, being some of the only individuals at their site to prescribe, felt unsupported in doing so. This highlights the notion that while some clinicians may be willing and trained to initiate BUP, organizational factors can deter the adoption and maintenance of the intervention.

### Clinician training and support

Participants reported that at the clinician level, the availability of training and support influenced the initiation of BUP and utilizing the EMBED intervention. Successful adoption could be facilitated with training and support in the following key areas: dissemination of evidence supporting ED-initiated BUP, practical knowledge about how and when to initiate BUP, and on-demand support from local champions to guide BUP initiation. Participants also reported that knowledge of the evidence basis for ED-initiated BUP, as well as the evidence that informed the intervention’s development, motivated them to initiate BUP. Furthermore, while many viewed the X-waiver training as necessary and modestly helpful, clinicians also stated that they needed training focused on the practicalities of initiating BUP in the ED. While participants valued training and support regarding the evidence and logistics for ED-initiated BUP, they preferred a well-designed, intuitive CDS tool that did not require formal training.

#### Role of evidence (intervention—organizational characteristics)

Participants discussed how different forms of evidence influenced their willingness to treat people experiencing OUD, including a better understanding of the morbidity and mortality associated with OUD (in particular after a non-fatal overdose), [7] information about the safety and effectiveness of BUP, and evidence about the positive impact of CDS tools:

It was like 1 in 10 mortality for the next year or something like that. It was just a startling, surprising number. Anyway, a bigger number than what I had thought about previously. … [the evidence] was pretty eye-opening. Then that plus the safety data that they presented on the medication was pretty compelling. (Interviewee 22, Site 4)

Another participant linked data pertaining to the mortality associated with OUD to other conditions that are commonly managed in the ED for which they have effective treatments to decrease the mortality:

The data is pretty clear. If you come in with opiate-use disorder, particularly if you have an overdose, your chance of death in the next year is as high as the people I see with an NSTEMI, and so, if I could do something to decrease that mortality, we should be doing it. I think that that is part of our role just like getting diabetics started on medication. You see an uncontrolled diabetic, it’s your role to talk to them about what diabetes medications they should be on, so I think the same thing with buprenorphine if this is a medication that’s potentially gonna help them. (Interviewee 14, Site 3)

In addition to feeling motivated by the evidence supporting ED-initiated BUP, participants also reported that knowing the evidence that supported the creation of the EMBED intervention influenced their decision to use and continue using it. Participants described how knowing the CDS tool was developed by experts and integrated into the EHR made them feel comfortable using it because they felt that they would be providing the standard of care:

It’s what I think got me over the hump of feeling comfortable because [when] somebody gives you a protocol, it makes it easy. … Make this less obscure for me. Give me something where I can follow steps through and then I can do it… I know it’s been invented by people far smarter than me with expertise. I’m doing what other people have recommended... I feel like I’m doing standard of care stuff here because ‘it’s in the EHR, it’s gotta be okay.’ For me, I think it gave me that level of comfort in what I was doing. (Interviewee 15, Site 3)

#### Importance of practical and applied training (implementation and sustainability infrastructure)

Participants had mixed opinions regarding the usefulness of the formal DATA 2000 X-waiver training, which was necessary to prescribe BUP outside of the ED during the trial period. Participants recognized that it provided useful background information on OUD and BUP, particularly for clinicians who needed that background information. However, most participants indicated that the waiver training alone was not relevant to understand BUP initiation in the ED and advocated for more focused, practical training focused on BUP in the ED.

I don’t really think having an eight-hour training was helpful personally, even though that’s what was at one point required. I guess there’s a lot of things that we learn in med school… [w]e don’t really need to know. I kind of feel like that’s one thing about [buprenorphine]. We’re like, oh, yeah, I’ve learned a bunch of times that it’s like a partial agonist and why it can’t be abused and stuff like that, but that doesn’t really matter on a daily basis. What matters is that I’m familiar enough with it that I don’t feel comfortable prescribing it, even though I know it’s—I think it’s part of my job and I try my best. (Interviewee 18, Site 3)

Most participants either did not recall the initial training for the CDS tool or did not find it particularly necessary. Instead, clinicians preferred to have someone available, such as a local champion, during a shift to walk them through the process of using the CDS tool. Some participants stated that if the CDS tool is in the same place in the EHR as other tools, and was intuitive, then minimal training is needed. Many respondents discussed that the simplicity of the EMBED intervention was integral to their decision to use and continue using it:

I don’t think it’s complex to use at all… it was intuitive and easy … it was like you select these, and then it goes and prints and magically gets done, which is what you want… I thought the usability was actually good which is why I was an early adopter of it when it first rolled out at our place. (Interviewee 15, Site 3)

### Connecting patients to ongoing treatment

#### Availability of referral resources (external environment)

In addition to factors at the clinician and organizational levels, the external context for each ED influenced clinicians’ decisions to initiate BUP and utilize the intervention. Of these, the strongest external factor was the ability to link patients who were initiated on BUP in the ED to ongoing treatment for OUD. As EDs were preparing to implement the CDS tool, they focused on establishing linkages and connections to sites for ongoing treatment, dedicating personnel to facilitate referrals, and integrating this piece into the CDS tool.

Many participants explained that successfully connecting patients to ongoing treatment required knowledge of places in their community accepting patients for OUD treatment and having a process in place to connect patients with those treatment facilities. They often found this linkage essential for deciding whether to use the tool and/or prescribe BUP. Given that BUP initiation in the ED is typically meant to serve as a bridge to longer-term treatment, some participants felt that prescribing without accessible follow-up care was pointless; if the patient cannot access or does not receive follow-up care, the medication does not serve its intended purpose:

The barriers [are] that…you have to have a place to refer them to. We can’t provide [ongoing treatment] to them. I can give them all the information about why [BUP] is awesome and why you should do it, and how to do a COWS score and all this stuff, they bought all that. What they didn’t buy and the whole crux of all of this is that, it’s a bridge… to long-term therapy. If you don’t have a place to send them to that you know is gonna be reliable within the amount of time that you’re giving them this bridge medication, there’s no point in doing it really ‘cause they are just gonna relapse and start using their old opioids again when they start withdrawing from the [BUP] ‘cause they only got seven days, and they don’t have anywhere to go. (Interviewee 28, Site 5)

Clinicians described that one benefit of the CDS tool was the fact that it generated the appropriate referral order in the EHR to link a patient to ongoing care. The personnel that were responsible for connecting patients to clinics for ongoing treatment varied across sites and included peer support specialists, social workers, or grant-funded addiction counselors. Some of these personnel were co-located in the participating EDs with the ability to meet with patients in-person to help facilitate the linkage for ongoing care. Other sites had centralized services where a message was sent through the EHR to the person who was tasked with arranging follow-up care.

I think the special sauce for this is that it takes the stuff that needs to be done by a physician and helps that physician do it well. Everything else that doesn’t need a medical degree—and gives it to somebody without a medical degree. I would use the pathway—I would try and use it exactly as it is here. If someone were to start this, the key thing would be making sure that the back end of this—an energetic social worker and a place where the patient can reliably go—and we can find out if they made it there, and if they stayed there. (Interviewee 5, Site 1)

#### Positive reinforcement for ED initiation of BUP (intervention—organizational perspective)

In addition to being able to connect patients to treatment using the CDS tool, participants also described the importance of receiving positive reinforcement for ED initiation of BUP. For example, participants appreciated hearing outcome information about patients for whom they initiated BUP, such as learning if the patient had successfully filled a prescription, went to a follow-up appointment, or stayed in treatment. As one participant explained, their local champion took responsibility for providing patient feedback:

[Our local champion] would send out emails being like, ‘You started this person on buprenorphine. They filled it, and they’ve had a refill at 30 and 60 days, and they’re still in treatment.’ It was positive feedback that what you did really did make a difference. I think that, at least in the short term, knowing on a case-by-case basis that what you did really did take and changed the course of somebody’s disease was really powerful. (Interviewee 17, Site 3)

This outcome information helped encourage clinicians to continue prescribing BUP when they could see its benefits on their patients.

### Tailor implementation to local setting (implementation and sustainability infrastructure)

The ability to create local workflows and tailor the EMBED intervention to fit the resources, staffing, and other characteristics of each site was an important factor that influenced the adoption and maintenance of ED-initiated BUP. Implementation was different at each site, reflecting differences in resources, EHRs, staffing, and decisions on how best to implement the intervention. Participants reported tailoring the EMBED intervention implementation based on these factors. For example, one site that had social workers available in their ED modified the CDS tool usage and BUP initiation process to involve them. As one participant described, tailoring the tool this way made prescribing BUP easy:

If I had someone who, let’s say, was in early withdrawal, I would say during my evaluation, ‘Are you interested in considering treatments to get off of the opioids?’ If they said yes, then I think what happens is we can consult the social worker, who then spends more time talking with them to really see if it’s a good fit. There’s some labs and things we can order if they are gonna go down the pathway that are clearly specified for the COWS score and figuring out where they’re at and ordering medications. It’s all spelled out so I don’t have to think about it. (Interviewee 4, Site 1)

Similarly, a participant at another ED discussed having clinical informatics support available at the front end of the implementation process to ensure that BUP initiation was adapted for their site-specific initiation process. Specifically, this site already had a CDS care pathway platform integrated into their EHR, making it easier to integrate the EMBED CDS tool into this existing structure. Furthermore, this facilitated adoption, as clinicians were already accustomed to using this section of the EHR for other care pathways. In contrast, some participants reported impeded or delayed adoption of the intervention because the CDS tool was not tailored to fit their site-specific EHR workflow and needs. One participant spoke to the challenges of implementing the EMBED intervention at their site because they used a different EHR system than the one the care pathway was originally built upon:

[Implementing the EMBED intervention] took a long time for our site. I think us and [Institution 1] were probably the last two to get this up, because we had to build it from scratch… all of the tech stuff that they gave our site to help build the pathway was not useful in any way to our IT people because it was on a completely different system… I think that really turned out not to be helpful either because we were on a more updated [EHR system 2] than they were. The care pathway build looked different in our site than their site, and so we couldn’t really help each other in that regard that much. (Interviewee 28, Site 5)

## Discussion

This qualitative analysis expands on findings from its parent clinical trial which found that 44% of the clinicians in the intervention arm initiated BUP [20]. Specifically, we found that the adoption, implementation, and maintenance of the EMBED intervention and ED-initiated BUP were influenced by organizational culture and commitment, clinician training and support, the ability to connect patients to ongoing treatment, and the ability to tailor implementation to each ED. The primary advantages of the EMBED intervention were in decreasing clinician burden, reducing the complexity of BUP initiation, and, at some of the EDs, generating orders or referrals that would facilitate the linkage of patients to ongoing care. Previous studies have described clinician perspectives on barriers of ED initiation of BUP, [14, 32] which identified a tool like EMBED as a facilitator. However, the EMBED trial showed that a user-centered CDS alone was not able to produce the desired patient-level outcome. This study identifies the importance of a more comprehensive and multilevel set of interventions or implementation strategies to further increase the adoption of ED-initiated BUP.

During the period in which the EMBED trial occurred, DATA 2000 training was required to obtain an X-waiver to prescribe BUP after discharge from the ED, an external factor that has since changed. This study identified several key areas of knowledge that are important to clinicians, which may be of more relevance given the elimination of the X-waiver training. First, clinicians often cited the knowledge of the evidence surrounding BUP initiation as an important factor in their decision to adopt this practice. Second, clinicians wanted more training on some of the practical aspects of BUP initiation in the ED, such as determining patient eligibility, degree of withdrawal, and dose selection. Lastly, clinicians valued having a trusted colleague who could serve as a local champion to consult with when they begin initiating BUP in the ED. These findings suggest that the training, education, and support to clinicians should be sequenced accordingly and could be more effective than a single, intensive training.

At the organizational level, creating a culture where BUP initiation was viewed as the norm or standard of care was key. However, this cultural piece had to be coupled with the appropriate allocation of resources and clear communication to all members of the clinical team. The exact implementation of these elements varied across the sites but, in general, required setting the expectation that ED clinicians should consider BUP initiation as part of their scope of practice, supporting them in receiving the necessary training and creating a workflow that is tailored to the staffing at each site. Engaging non-clinician staff members in the process was important, as they often had the first contact with the patient and needed to know that BUP initiation was an option within their ED. Furthermore, social workers, care coordinators, or peer support specialists were integral in conducting a more in-depth assessment and connecting patients to ongoing treatment.

This study is unique in that it evaluated the determinants of adoption, implementation, and maintenance of both the EMBED CDS intervention and the broader practice of ED-initiated BUP. This allows for a deeper understanding of how the EMBED intervention can be leveraged to enhance the adoption of ED-initiated BUP, while also understanding what other implementation components and strategies may be needed beyond CDS. There were several limitations to this study. First, while we were able to identify factors that participants believed influenced their decision to use the CDS tool and initiate BUP, we were not able to prospectively assess whether addressing these factors would increase adoption. A key next step will be to develop and test implementation strategies informed by this study’s findings. Additionally, this study recruited participants who were familiar with the CDS tool from within the original EMBED trial sites to understand the influence of the CDS tool on BUP initiation. Thus, perspectives from participants who were not familiar with the CDS tool were not represented in this analysis. Because all participants were familiar with the CDS tool, the sample may reflect clinicians who were more likely to initiate BUP or adopt a CDS intervention in general. Another limitation that may affect generalizability is that the majority of our sample was physicians, with only a few physician assistants and no nurse practitioners or other clinical staff included in the sample. Additionally, while there was a mixture of participants from both academic and community EDs, all the EDs were nested within academic healthcare systems. Lastly, given that our goal was to understand the determinants of adoption, implementation, and maintenance of the EMBED intervention across all participating sites, these findings do not reflect a formal comparison between sites. Future research is recommended to better understand how contextual differences between sites affect implementation. While this may impact the generalizability of these findings, it also highlights the need to carefully tailor future implementation of the EMBED intervention and ED-initiated BUP as these innovations scale out.

As in previous studies, [32, 33] participants in this study identified the ability to link patients to ongoing care as a critical barrier to initiating. From a harm reduction perspective, linkage to ongoing treatment should not be considered a requirement for ED-initiated BUP [34]. Regardless, all sites in this study had a process in place to connect patients with ongoing treatment, though access varied based on availability in the surrounding local community. This paper builds upon prior work to provide key suggestions for how to implement ED initiation of BUP at other EDs. Each department was given latitude to determine their own workflow and delegate this task to the appropriate team member at each institution. Then, the CDS tool could help by generating the referral in the EHR to facilitate this linkage. This highlights how the CDS tool can be customized to each institution to assist with the process of linking patients for ongoing care.

Previous research on ED-initiated BUP indicated that clinicians desired a protocol or pathway that was ideally within the EHR to facilitate the process of BUP initiation [14]. This study confirms that finding; participants viewed the EMBED intervention favorably, and characteristics of the EMBED intervention were not cited as barriers to BUP initiation.

The EMBED intervention can help clinicians initiate BUP in the ED, but successful implementation in the ED setting requires attention to other, multilevel factors. To identify those determinants, the PRISM framework was leveraged in this study to inform the development of the interview guide as well as the selection of a priori codes for analysis. This approach provided a structure by which to investigate the multilevel factors that influenced the use of the EMBED intervention. In turn, these determinants can be used to develop implementation strategies that should enhance the adoption, implementation, and maintenance of ED-initiated BUP. Table 3 provides a list of potential implementation strategies to address the determinants identified in this study, organized by PRISM domain and corresponding conceptual category from the Expert Recommendations for Implementing Change (ERIC) project’s compilation of implementation strategies [35]. Our study demonstrated that the ability to tailor implementation to each ED was an important factor. Thus, these potential implementation strategies could be viewed as a menu that leaders, administrators, and clinicians in the ED could review when deciding how best to implement BUP initiation in their local setting. There are several approaches that can be used to accomplish this goal [36].

**Table 3:** Multilevel implementation strategies to support ED initiation of buprenorphine through the integration of the EMBED clinical decision support tool, grouped by PRISM domain. Each strategy was also placed within the corresponding conceptual category according to the groupings from the ERIC project [35]

PRISM domain	Implementation strategy (ERIC category)
**Intervention (organizational perspective)**	• Tailor intervention to the local environment (adapt and tailor to context) • Low complexity/intuitive CDS tool (support clinicians) • Observability—provide feedback to clinicians when patients have positive outcomes, both in the short term (e.g., improved symptoms while in the ED) and long term (e.g., patient establishes care for OUD treatment after discharge) (use evaluative and iterative strategies) • Identify existing interdisciplinary team members in the ED and the organization to implement the intervention and develop workflows to facilitate coordination and completion of key tasks (support clinicians)
**Recipients (organizational characteristics)**	• Create policies and best practices that prioritize patients with OUD (change infrastructure) • Organizational support for interdisciplinary team members to implement the intervention in the ED and organization (support clinicians) • Incentives/mandates for X-waiver (change infrastructure) • Develop and support local champion(s) (develop stakeholder interrelationships)
**Recipients (patient characteristics)**	• Resources and counseling for patients on options (including harm reduction strategies) when patients are not ready to initiate buprenorphine (engage consumers)
**Implementation and sustainability infrastructure**	• Sufficient training for prescribing clinicians (train and educate stakeholders) • IT/EHR support (change infrastructure) • Routine monitoring and feedback on both CDS use and buprenorphine initiation (use evaluative and iterative strategies)
**External environment**	• Develop partnerships to refer a patient for ongoing OUD treatment (develop stakeholder interrelationships)

The optimal delivery, amount, and type of training required for an ED clinician to undertake BUP initiation requires further research, particularly as training is no longer required as part of the process of obtaining an X-waiver for emergency physicians. While our study identified many factors that influenced clinicians’ decisions to initiate BUP, understanding the prevalence of these factors and prospective testing of implementation strategies that target these determinants is needed.

## Conclusion

This study identifies several key determinants that influence the adoption, implementation, and maintenance of a CDS tool that has been shown to increase the adoption of ED-initiated BUP. In turn, these determinants can serve as targets for multilevel implementation strategies that can help to scale both the CDS tool and the practice of initiating BUP in the ED.

## Supplementary Information

Additional file 1: Implementation Guide to incorporate the EMBED Clinical Decision Support Tool for Emergency Department Buprenorphine Initiation

## Data Availability

Data sharing is not applicable to this article as no datasets were generated or analyzed during the current study as the study was a qualitative study.

## Abbreviations

BUP: Buprenorphine
CDS: Clinical decision support
DATA: Drug Addiction Treatment Act
ED: Emergency department
EHR: Electronic health record
EMBED: EMergency department-initiated BuprenorphinE for opioid use Disorder
ERIC: Expert Recommendations for Implementing Change
OUD: Opioid use disorder
PRISM: Practical, Robust Implementation, and Sustainability Model
