# Supplementary material for: Implementation strategies to address the determinants of adoption, implementation, and maintenance of a clinical decision support tool for emergency department buprenorphine initiation: a qualitative study
Source: Implement Sci Commun. 2023 Apr 20;4:41. doi: 10.1186/s43058-023-00421-7 (PMC10117277; doi:10.1186/s43058-023-00421-7)
Supplement: Supplementary file 1 — Implementation Guide to incorporate the EMBED Clinical Decision Support Tool for Emergency Department Buprenorphine Initiation [file 43058_2023_421_MOESM1_ESM.docx]

**Implementation Guide to incorporate the EMBED Clinical Decision Support Tool for Emergency Department Buprenorphine Initiation**

Background: The emergency department (ED) represents a key opportunity to engage patients in evidence-based treatment for opioid use disorder (OUD), including buprenorphine. Buprenorphine can be safely initiated in the ED, is cost-effective, and is associated with a nearly two-fold increase in the probability of remaining engaged in formal addiction treatment following discharge.^1, 2^ However, ED-initiated buprenorphine remains underutilized.^3^ The [EMBED (EMergency department-initiated BuprenorphinE for opioid use Disorder) clinical decision support (CDS)](https://embed.ynhh.org/) is a clinician-facing application that was developed through a user-centered design process to support emergency medicine clinicians in the initiation of buprenorphine for the treatment of OUD in the emergency department.^4, 5^ In-depth interviews were conducted with 28 clinicians across 5 healthcare systems to understand how to best incorporate buprenorphine initiation through the EMBED CDS into routine practice. This guide is intended for leaders, administrators, and clinicians in EDs and healthcare systems to assist with incorporating buprenorphine initiation for patients with OUD into the ED workflow.

**Recommendations (what to do):**

1. Develop a culture where buprenorphine initiation is the standard of care. EDs that were successful in incorporating buprenorphine initiation into their clinical workflows had a shared belief among clinicians and staff members that caring for patients with OUD was a responsibility shared across the clinical team.
2. Commit time and resources to support patients with OUD in the ED. Designating at least one clinical champion within the ED was viewed as very helpful for clinicians who had less experience with buprenorphine initiation. Existing staff members, such as behavioral health providers, social workers, or peer support specialists, may need to take on new roles as well, in which case it is important to support them in taking on additional work.
3. Recognize that buprenorphine in the ED initiation is an evidence-based best practice that is safe and effective. Multiple studies indicate the buprenorphine initiation in the ED is more effective than other interventions, such as SBIRT, for engaging and retaining people with OUD in treatment.
4. Train clinicians on practical aspects of buprenorphine initiation in the ED. It is important to couple trainings related to buprenorphine initiation with practical tips for buprenorphine initiation that are specific to their ED.
5. Develop a workflow to connect patients to ongoing treatment after discharge from the ED. Every ED does this slightly differently based on staffing models within the ED and connections to practices in the community. The important piece is to have a system in place where patients who are started on buprenorphine from the ED can successfully establish with ongoing treatment within a reasonable timeframe (typically 5-7 days).
6. Provide positive feedback to clinicians after initiating buprenorphine. Particularly when starting anew, clinicians appreciate hearing about patients who successfully established care for ongoing treatment after starting buprenorphine from the ED. When possible, try to capture this information and provide feedback to the clinicians.
7. Adapt and tailor workflows to your EDs and healthcare system. Successful implementation and sustainment of buprenorphine initiation from the ED requires many aspects of the process to be tailored to each department’s IT infrastructure, staffing, and resources.

**Recommended implementation strategies (how to do it):**

Clinician level:

Review evidence supporting buprenorphine initiation in the ED.

- Buprenorphine can be safely initiated in the ED.
- Initiating buprenorphine from the ED significantly improves the likelihood that a patient with OUD will engage in formal addiction treatment following discharge.

Practical training for buprenorphine initiation.

- While the DATA 2000 waiver (X waiver) no longer exists, practitioners with DEA licenses will be required to complete 8 hours of training related to substance use disorder when they renew their DEA license. Details regarding this change will be forthcoming in June 2023.
- In addition to formal training related to substance use disorders, clinicians appreciated training that was relevant to how to initiate buprenorphine in their specific ED.

Provide positive feedback to clinicians after initiating buprenorphine. This can reinforce the new behavior and can be accomplished in several ways:

- Create reports that show how many patients are being started on buprenorphine in the ED by each clinician.
- When initiating buprenorphine on a patient in the ED, provide feedback to the clinician when patient’s status/symptoms improve.
- Track patient after discharge to see how many established for ongoing treatment to demonstrate the effect of the intervention.

Organizational level:

Develop a culture where buprenorphine initiation is the norm.

- Set the expectations that all clinicians in the emergency department will be able to initiate buprenorphine when indicated.
- Create incentives for clinicians to receive training on the treatment of OUD in the ED.
- Clear communication to nursing staff, behavioral health, social work, and other clinical staff that buprenorphine is an option for patients with OUD.

Commit time and resources to support patients with OUD in the ED.

- Identify and support clinical champion(s) that can support other clinicians.
- Designate key tasks to appropriate staff members. For example:
  - RNs screen patients and calculate COWS scores,
  - Behavioral health or social work assess patient's readiness for treatment,
  - Clinician orders buprenorphine,
  - Social work, care manager, or peer support specialist can arrange for follow up.

Adapt and tailor workflows to your EDs and healthcare system.

- Integrate the EMBED CDS into workflows for buprenorphine initiation. This can be accomplished through integration within the EHR, using [smartphone apps](https://apps.apple.com/us/app/bup-initiation/id1574350314#?platform=iphone?utm_source=starter&utm_medium=start-page&utm_campaign=minimal-starter), or use of [MDCalc](https://www.mdcalc.com/emergency-department-initiated-buprenorphine-opioid-use-disorder-embed).
- Tailor workflow to existing resources and staff within each ED.

External environment:

Work with institution and community partners to develop a workflow to connect patients to ongoing treatment after discharge from the ED.

- Identify clinics in your catchment area that prescribe medications for OUD and understand their requirements.
- Identify staff members who can work with treatment partners and help patients coordinate appointments to receive ongoing care for OUD.

Citations

1. Busch SH, Fiellin DA, Chawarski MC, Owens PH, Pantalon MV, Hawk K, et al. Cost-effectiveness of emergency department-initiated treatment for opioid dependence. Addiction. 2017;112(11):2002-10.

2. D'Onofrio G, O'Connor PG, Pantalon MV, Chawarski MC, Busch SH, Owens PH, et al. Emergency department-initiated buprenorphine/naloxone treatment for opioid dependence: a randomized clinical trial. JAMA. 2015;313(16):1636-44.

3. Martin SA, Chiodo LM, Bosse JD, Wilson A. The Next Stage of Buprenorphine Care for Opioid Use Disorder. Ann Intern Med. 2018;169(9):628-35.

4. Melnick ER, Holland WC, Ahmed OM, Ma AK, Michael SS, Goldberg HS, et al. An integrated web application for decision support and automation of EHR workflow: a case study of current challenges to standards-based messaging and scalability from the EMBED trial. JAMIA Open. 2019;2(4):434-9.

5. Melnick ER, Nath B, Dziura JD, Casey MF, Jeffery MM, Paek H, et al. User centered clinical decision support to implement initiation of buprenorphine for opioid use disorder in the emergency department: EMBED pragmatic cluster randomized controlled trial. BMJ. 2022;377:e069271.
